# Alterations of the expression levels of glucose, inflammation, and iron metabolism related miRNAs and their target genes in the hypothalamus of STZ-induced rat diabetes model

**DOI:** 10.1186/s13098-022-00919-5

**Published:** 2022-10-10

**Authors:** Edina Pandur, István Szabó, Edina Hormay, Ramóna Pap, Attila Almási, Katalin Sipos, Viktória Farkas, Zoltán Karádi

**Affiliations:** 1 Department of Pharmaceutical Biology, Faculty of Pharmacy, University of Pécs, Rókus u. 4., 7624 Pécs, Hungary; 2 Institute of Physiology, Medical School, University of Pécs, Szigeti út 12., 7624 Pécs, Hungary; 3 Institute of Pharmaceutical Chemistry, Faculty of Pharmacy, University of Pécs, Rókus u. 4., 7624 Pécs, Hungary

**Keywords:** Hypothalamus, Iron metabolism, Diabetes, Fractalkine, Inflammation, miRNA

## Abstract

**Background:**

The hypothalamus of the central nervous system is implicated in the development of diabetes due to its glucose-sensing function. Dysregulation of the hypothalamic glucose-sensing neurons leads to abnormal glucose metabolism. It has been described that fractalkine (FKN) is involved in the development of hypothalamic inflammation, which may be one of the underlying causes of a diabetic condition. Moreover, iron may play a role in the pathogenesis of diabetes via the regulation of hepcidin, the iron regulatory hormone synthesis. MicroRNAs (miRNAs) are short non-coding molecules working as key regulators of gene expression, usually by inhibiting translation. Hypothalamic miRNAs are supposed to have a role in the control of energy balance by acting as regulators of hypothalamic glucose metabolism via influencing translation.

**Methods:**

Using a miRNA array, we analysed the expression of diabetes, inflammation, and iron metabolism related miRNAs in the hypothalamus of a streptozotocin-induced rat type 1 diabetes model. Determination of the effect of miRNAs altered by STZ treatment on the target genes was carried out at protein level.

**Results:**

We found 18 miRNAs with altered expression levels in the hypothalamus of the STZ-treated animals, which act as the regulators of mRNAs involved in glucose metabolism, pro-inflammatory cytokine synthesis, and iron homeostasis suggesting a link between these processes in diabetes. The alterations in the expression level of these miRNAs could modify hypothalamic glucose sensing, tolerance, uptake, and phosphorylation by affecting the stability of hexokinase-2, insulin receptor, leptin receptor, glucokinase, GLUT4, insulin-like growth factor receptor 1, and phosphoenolpyruvate carboxykinase mRNA molecules. Additional miRNAs were found to be altered resulting in the elevation of FKN protein. The miRNA, mRNA, and protein analyses of the diabetic hypothalamus revealed that the iron import, export, and iron storage were all influenced by miRNAs suggesting the disturbance of hypothalamic iron homeostasis.

**Conclusion:**

It can be supposed that glucose metabolism, inflammation, and iron homeostasis of the hypothalamus are linked via the altered expression of common miRNAs as well as the increased expression of FKN, which contribute to the imbalance of energy homeostasis, the synthesis of pro-inflammatory cytokines, and the iron accumulation of the hypothalamus. The results raise the possibility that FKN could be a potential target of new therapies targeting both inflammation and iron disturbances in diabetic conditions.

**Graphical Abstract:**

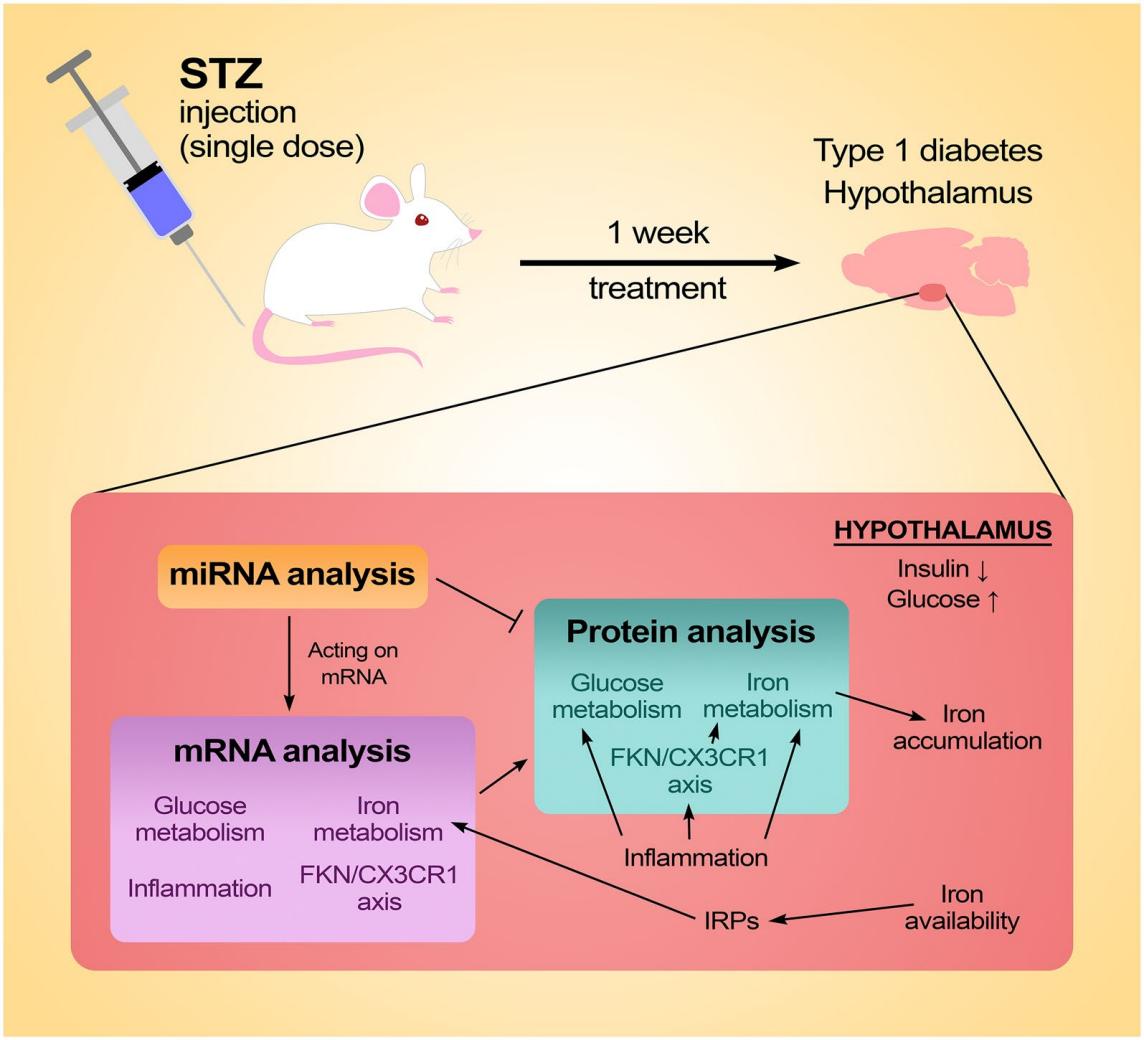

**Supplementary Information:**

The online version contains supplementary material available at 10.1186/s13098-022-00919-5.

## Background

Diabetes, a systemic metabolic disease affects people all over the world. Diabetes can arise from different metabolic dysfunctions like insulin resistance, glucose intolerance, or from obesity [1]. In recent decades it has been revealed that the central nervous system (CNS), especially the hypothalamus is implicated in the development of diabetic conditions [2, 3]. The hypothalamic neurons in the nuclei (e.g. arcuate, ventromedial) respond to hyper- or hypoglycaemia to maintain circulating glucose concentration [4, 5]. Dysregulation of the hypothalamic glucose-sensing neurons leads to abnormal glucose metabolism [6].

The alterations of the neuron-glial cell interactions were also observed in the case of diet-induced metabolic changes and may contribute to the establishment of diabetes [7]. Fractalkine (FKN)/fractalkine receptor (CX3CR1) axis is crucial in the regulation of microglia, the immune cells of CNS [8]. FKN/CX3CR1 regulates the expression of pro-inflammatory cytokines (IL-1β, IL-6, and TNF-α) promoting neuroinflammation. It has been described that FKN is involved in the development of hypothalamic inflammation, which may be one of the underlying causes of the disruption of energy balance [9, 10].

Iron may play a role in the pathogenesis of diabetes. Iron overload of the human body increases the risk of the establishment of metabolic disorders [11, 12]. Recently, it has been revealed that the serum level of the iron regulatory hormone hepcidin and ferritin correlates with the energy balance markers such as leptin [13, 14], which functions as a regulator of glucose-sensing neurons in the hypothalamus [15]. Moreover, insulin influences iron metabolism by affecting the transport of transferrin receptor 1 (TfR1) into the cell membranes and increasing ferritin (FTH) synthesis [16]. On the other hand, iron metabolism is regulated by inflammation as well as by FKN, which can increase the iron absorption of neurons and inhibit iron release through the receptor of hepcidin, the iron exporter ferroportin (FP) [17, 18].

The molecular mechanisms with which the glucose metabolism, inflammatory processes, and iron homeostasis of hypothalamus crosstalk, are still under investigation. The role of small non-coding RNA molecules, the miRNAs have been investigated in metabolic disorders [19]. The miRNAs affect the stability of the mRNA molecules and mainly inhibit their translation by decreasing the rate of protein synthesis of the target genes [20]. Numerous miRNAs have been described as the regulators of energy balance and iron homeostasis [21–24].

In the present study, we investigated the molecular biological background and the possible links between diabetes, inflammation, and iron metabolism in the hypothalamus of an STZ-induced rat type 1 diabetes model.

Using the miRNA array, we analysed the expression of diabetes, inflammation, and iron metabolism related miRNAs. Determination of the effect of miRNAs altered by STZ treatment on the target genes was carried out at protein level.

Based on the findings, it is supposed that glucose metabolism, inflammation, and iron homeostasis of the hypothalamus are linked via the altered expression of common miRNAs as well as the increased expression of FKN, which contribute to the imbalance of energy homeostasis, the synthesis of pro-inflammatory cytokines and the iron accumulation and retention of the hypothalamus.

## Methods

### Animals and treatments

Seven-week-old, male Wistar rats (*Rattus norvegicus*) were purchased from Janvier Labs (Le Genest-Saint-Isle, France). After one week of resting, the animals were randomly separated into two groups: Streptozotocin treated (n = 18) and control (n = 18). A total of 36 animals were used in the experiments. Streptozotocin (STZ; Merck Life Science Kft., Budapest, Hungary) was solved in citrate buffer (50 mmol/L, pH 4.5). The single dose of STZ was determined by body weight (65 mg/bwkg) and was injected i.v. into the tail vein for developing type 1 diabetes The control animals received a single i.v. injection of citrate buffer according to their body weight. Hyperglycaemia was determined by measuring the blood sugar concentration from the tail vein using the Accu-Chek blood sugar meter (Roche Hungary Kft., Budapest, Hungary) after one week of injection. Rats were considered diabetic if the peripheral blood glucose concentration was higher than 20 mmol/L after one week of STZ treatment. Animals were kept under SPF conditions under permits BAI/35/90–5/2019, BA/35/77-2/2019, and BA/35/77-2/2020 (issued by the Baranya County Government Office). The Institutional Animal Care and Use Committee has approved the animal use protocols (BAI/35/90–5/2019, BA/35/77-2/2019, and BA/35/77-2/2020). All applicable international, national, and/or institutional guidelines for the care and use of animals were followed. No mortality was observed and therefore no animals were excluded.

### Dissection of the hypothalamus

The animals were sacrificed after one week of STZ treatment, the whole brain was removed, and the hypothalamus was dissected from each animal. The area of the hypothalamus was identified according to the stereotaxic rat brain atlas of Paxinos and Watson [25]. Firstly, the diencephalon was dissected by two coronal sections, the anterior section was made at the rostral of the optic chiasm (between Bregma and B + 0.5 mm) and the posterior cut at the caudal end of mammillary bodies (between B-5.6 and B-6 mm). In the second step, a 3 × 3 mm area was removed at the ventral-medial part of each side of sections to separate the hypothalamus from the surrounding areas.

### miRNA isolation and miRNA PCR assay

Total miRNA was isolated from the hypothalamus tissue samples of 6 STZ-treated and 6 control animals using a miRPremier miRNA isolation kit (Merck Life Technologies Kft., Budapest, Hungary). miRNA samples were pooled into STZ-treated and control groups. 250 ng of pooled miRNA of both groups was applied for cDNA synthesis using miScript II RT Kit (Merck Life Sciences Kft., Budapest, Hungary). The mature miRNA expression profiling was performed with miScript miRNA PCR Assay for Rat diabetes using SYBR Green-based real time PCR protocol (Qiagen GmbH, Hilden, Germany). miScript miRNA PCR Array data analysis was performed using Qiagen excel template for miScript miRNA PCR Array (www.qiagen.com) and (∆∆Ct) method, the expression levels of miRNAs were determined as fold change compared to the untreated control. All kits were used according to the manufacturers’ protocols.

### Real time PCR

Total RNA was isolated from the hypothalamus tissue samples of 12 animals (6 animals from each group) using Aurum Total RNA Mini Kit (Bio-Rad Inc., Hercules, CA). Complementary DNA was synthesised from 100 ng of total RNA using IScript Select cDNA Synthesis Kit (Bio-Rad Inc., Hercules, CA) according to the manufacturer’s protocol. Determination of gene expressions was performed in a CFX96 One Touch Real-Time System (Bio-Rad Inc., Hercules, CA) applying iTaq™ Universal SYBR® Green Supermix (Bio-Rad Inc., Hercules, CA). The total reaction volume was 20 µL. The specificity of the reactions was determined by generating a melting curve after each run. Relative quantification was carried out by the Livak (∆∆Ct) method using the Bio-Rad CFX Maestro software 1.2. (Bio-Rad Inc., Hercules, CA). β-actin housekeeping gene was used for normalization in each sample. The relative expression of the untreated samples was considered as 1. The mRNA expression of the STZ-treated samples was compared to the untreated samples and was expressed as fold change. The primer sequences used in this study are described in Table 1.

**Table 1:** Real time primer list

Primer	Sequence 5′ → 3′
BMPR forward	GGGATTGGTGAGAGTCGAA
BMPR reverse	TTTCACAAGATTGATGTCCCC
CX3CL1 forward	TGACTGGATCGTCTCCCTCA
CX3CL1 reverse	CGGCCAAATGGTGGTAGAGA
CX3CR1 forward	TCTTCCTCTTCTGGACGCCT
CX3CR1 reverse	CTAAACGCCACTGTCTCCGT
DMT-1 forward	TGTTCTACTTGGGTTGGCAGT
DMT-1 reverse	ATTGCCACCGCTGGTATCTT
FECH forward	GGCTGTCCCGGAAATGCTT
FECH reverse	GCCATACACCAGCGGCTC
FP forward	ACGGAAACAGCCTCCTCTTG
FP reverse	CGTCTGGGCCACTTTAAGTCT
FXN forward	TGTCTCTTTTGGGGATGGCG
FXN reverse	CTTCCCGGTCCAGTCATAGC
FTH forward	CCTACGTCTATCTGTCCATGTCTT
FTH reverse	TGGTTCTGCAGCTTCATCAGT
FTL forward	AGTTGCAGAACGAACGC
FTL reverse	GTTTTACCCCACTCATCTTGA
FTMT forward	GGAAGCGAGAGCAAGCACTA
FTMT reverse	ACTCCATCCAGGTCTTGGGA
GAPDH forward	TGATGGGTGTGAACCACGAG
GAPDH reverse	TCATGAGCCCTTCCACGATG
GCK forward	CTCTGGGCACCAACAAATGC
GCK reverse	GTTCATGTGCCCGTTGTGAG
GLUT4 forward	CACAAGGCACCCTCACTACC
GLUT4 reverse	GGAGGAAATCATGCCACCCA
HAMP forward	CTATCTCCGGCAACAGACGA
HAMP reverse	GGGAAGTTGGTGTCTCGCTT
HXK-2 forward	ATCGCCTGCTTATTCACGGA
HXK-2 reverse	TGGTAGCTCCTAGCCCTTTCT
IGFR1 forward	CTCTAAGGCCAGAGGTGGAGAATA
IGFR1 reverse	TGTGGACGAACTTGTTGGCAT
IL-1β forward	AAATGCCTCGTGCTGTCTGA
IL-1β reverse	AGGCCACAGGGATTTTGTCG
IL-6 forward	ACCCCAACTTCCAATGCTCTC
IL-6 reverse	ATGGTCTTGGTCCTTAGCCAC
INSR forward	CAACAACAAGTGCATCCCCG
INSR reverse	TCTCGCCTTCGAGGATTTGG
Lactoferrin forward	TGAAGGACGCCACTGTCTTG
Lactoferrin reverse	CAAGGCACAACAGCTCGAAG
LEPR forward	TTGTTGTGAAGCCCGATCCA
LEPR reverse	GCTTTTGTTTGGCTGTCCCA
NFS-1 forward	CAGCAGGTAGCGTCTCTGAT
NFS-1 reverse	AGAACCTGGCCACTCCCTTA
PEPCK forward	GGGGGTGTTTACTGGGAAGG
PEPCK reverse	CGGTTCCTCATCCTGTGGTC
TfR1 forward	TGCTTCAGAGTGCTCCCTTG
TfR1 reverse	GACAATGGCTCCCCTCCAAA
TNF-α forward	CTCAAGCCCTGGTATGAG
TNF-α reverse	CCTCAAAGTAGACCTGCCCG
TGFβ forward	CTGCTGACCCCCACTGATAC
TGFβ reverse	AGCCCTGTATTCCGTCTCCT
β-actin forward	CCCGCGAGTACAACCTTCTT
β-actin reverse	TCATCCATGGCGAACTGGTG

### Western blot analysis

The hypothalamus tissue samples (6 animals from each group) were lysed in 150 µL of ice-cold lysis buffer (50 mmol/L Tris–HCl, pH 8, 150 mmol/L NaCl, 0.5% sodium deoxycholate, 1% Triton-X 100, 0.1% SDS) containing complete mini protease inhibitor cocktail (Roche Ltd., Basel, Switzerland). The protein contents of the samples were determined using a DC Protein Assay Kit (Bio-Rad Laboratories, Hercules, CA, USA). Protein samples were separated into 10% or 12% polyacrylamide gels. The gels were transferred by electroblotting to nitrocellulose membranes (Pall AG, Basel, Switzerland). The following polyclonal rabbit antibodies were used for WB experiments: anti-GLUT4 IgG (1:1000; Bio-Techne, Minneapolis, MN, USA), anti-insulin receptor (INSR) IgG (1:1000; Thermo Fisher Scientific Inc., Waltham, MA, USA), anti-hexokinase 2 (HXK-2) IgG (1:1000; Bio-Techne, Minneapolis, MN, USA), anti-glucokinase (GCK) IgG (1:1000; Bio-Techne, Minneapolis, MN, USA), anti-glyceraldehyde 3-phosphate dehydrogenase (GAPDH) IgG (1:1000; Merck Life Science Kft., Budapest, Hungary), anti-leptin receptor IgG (1:1000; Thermo Fisher Scientific Inc., Waltham, MA, USA), anti-fractalkine (CX3CL1) IgG (1:1000; Thermo Fisher Scientific Inc., Waltham, MA, USA), anti-fractalkine receptor (CX3CR1) IgG (1:1000; Thermo Fisher Scientific Inc., Waltham, MA, USA), anti-ferroportin (Fp) IgG (1:1000; Bio-Techne, Minneapolis, MN, USA), anti-divalent metal transporter 1 (DMT-1) IgG (1:1000; Thermo Fisher Scientific Inc., Waltham, MA, USA) anti-transferrin receptor 1 (TfR1) IgG (Thermo Fisher Scientific Inc., Waltham, MA, USA), anti-ferritin light (FTL) chain IgG (Thermo Fisher Scientific Inc., Waltham, MA, USA), anti-mitochondrial ferritin (FTMT) IgG (1:1000, Thermo Fisher Scientific Inc., Waltham, MA, USA), anti-ferrochelatase (FECH) IgG (1:1000; (Thermo Fisher Scientific Inc., Waltham, MA, USA), anti-NFS-1 IgG (Thermo Fisher Scientific Inc., Waltham, MA, USA). All these primary antibodies were used for 1 h incubation at room temperature. Ferritin heavy chain (FTH) IgG (1:1000; Cell Signaling Technology Europe, Leiden, the Netherlands) was used overnight at 4 °C. Anti-β-actin IgG (1:2000; Merck Life Science Kft., Budapest, Hungary) was used as loading control of the Western blots. Horseradish peroxidase (HRP)-linked goat anti-rabbit IgG was used (1:3000; Bio-Rad Laboratories, Hercules, CA, USA) as the secondary antibody, for 1 h at room temperature. Western blot development was performed with traditional colourimetric detection using Fuji medical X-ray film (Fujifilm Corporation, Tokyo, Japan) and WesternBright ECL chemiluminescent substrate (Advansta Inc., San Jose, CA, USA). Optical densities of the protein bands were analyzed by ImageJ software [26]. The results were expressed as a percentage of the target protein/β-actin ratio.

### Statistical analysis

The miRNA PCR assay was performed in triplicate. Real time PCR determinations were carried out in triplicate in three independent experiments. Western blots are representative of three independent experiments. Statistical analysis was performed using SPSS software (IBM Corporation, Armonk, NY, USA). Statistical significance was determined by the Student’s t-test. Data are shown as mean ± standard deviation (SD). The results were considered statistically significant at a *p*-value < *0.05*.

## Results

### Animal parameters showing hyperglycaemia

One week after the treatments, the blood sugar concentrations of the animals in the control group and the STZ group were 6.95 ± 1.05 mmol/L (n = 18), and 27.09 ± 5.29 mmol/L (n = 18), respectively showing the development of hyperglycaemia. The body weight of the control animals was 260 ± 10.14 g at the beginning of the experiment and 311.21 ± 12.25 g at the end of the experiment. The body weight of the animals was 258 ± 11.2 g before the STZ treatment and 272 ± 11.81 g after one weak STZ treatment showing a reduced rate of body weight gain. The relative mRNA expression level of phosphoenolpyruvate carboxykinase (PEPCK) was significantly increased in the hypothalamus of STZ-treated animals to 14.58 (± 2.52) compared to the expression level of the control, which was considered as 1, showing disturbance in the gluconeogenesis, which is a sign of *diabetes mellitus*. On the other hand, the relative mRNA expression of glyceraldehyde 3-phosphate dehydrogenase (GAPDH), a glycolytic enzyme contributing to diabetic symptoms significantly decreased to 0.53 ± 0.06 in the STZ-treated rat hypothalamus compared to the control mRNA level, which was regarded as 1.

The miRNAs regulating glucose metabolism show changes in the STZ-treated rat hypothalamus and modify the protein levels of the target genes.

**Fig. 1:**
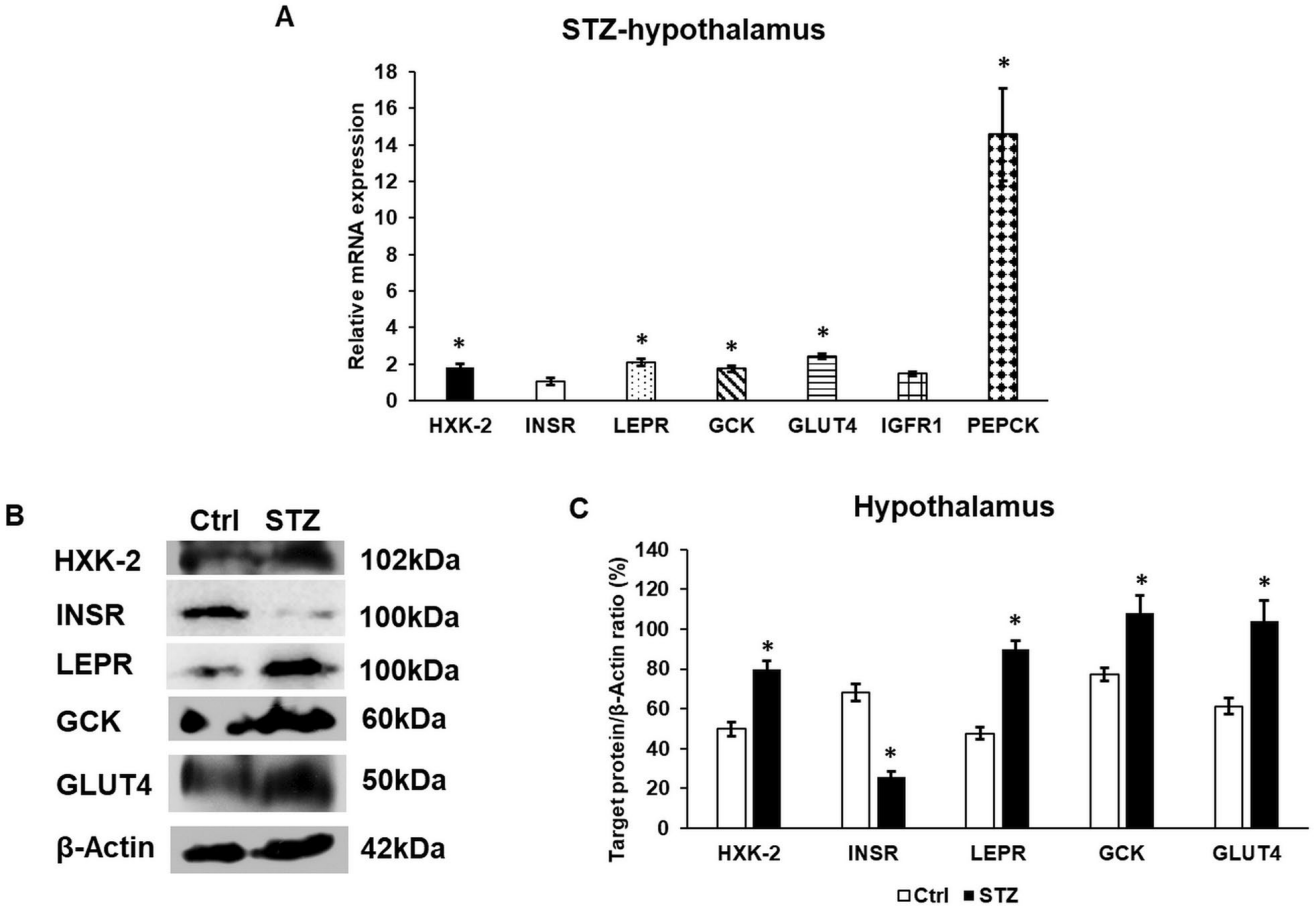
mRNA (**A**) and Western blot (**B**, **C**) analyses of the target genes of glucose metabolism related miRNAs. Real time PCR was carried out using a SYBR Green protocol. β-actin housekeeping gene was used as the internal control for the normalization of the reaction. The relative gene expression of the control hypothalamus samples was regarded as 1. Real time polymerase chain reactions were carried out in triplicate in each independent experiment. For the WB, the same amount of protein from each lysate was used for the analysis. **A** mRNA expression levels of target genes. **B** Western blot analysis of the target proteins. **C** Optical density analysis of the target proteins. The columns represent mean values, error bars show the standard deviation (SD) of three independent determinations (n = 3). Asterisk indicates *p* < *0.05* compared to the control. HXK-2: hexokinase-2; INSR: insulin receptor; LEPR: leptin receptor; GCK: glucokinase; GLUT4-glucose transporter 4; IGFR: insulin-like growth factor receptor 1; PEPCK: phosphoenolpyruvate carboxykinase; STZ: streptozotocin

We examined the relative expression of 88 rno-miRNAs with miRNA PCR Assay. According to the significance of clinical aspects, 18 miRNAs were chosen for further investigation. The miRNAs related to glucose metabolism showing alteration in the expression rate at STZ treatment are listed in Table 2. The changes in the miRNA expression levels suggest the modification of the mRNA expression rates of their target genes in the STZ-treated animals. Therefore, the relative mRNA expression of GLUT4, hexokinase-2 (HXK-2), insulin receptor (INSR), leptin receptor (LEPR), glucokinase (GCK), insulin-like growth factor 1 receptor (IGFR1) and PEPCK was determined by real time PCR (Fig. 1A).

**Table 2:** Changes in the relative expression of miRNAs related to glucose metabolism in the STZ-treated rat hypothalamus

miRNA	Expression (± SD)	Target genes
rno-miR-135b-5p	1.791 (0.074)	GLUT4
		Hexokinase-2
rno-miR-194-5p	1.539 (0.051)	Insulin receptor
rno-miR-21-5p	2.466 (0.082)	GLUT4
rno-miR-200a-3p	0.051 (0.021)	Leptin receptor
		Insulin receptor
rno-miR-152-3p	0.517 (0.054)	Glucokinase
rno-miR-96-5p	0.064 (0.019)	IGF1R
rno-miR-29a-3p	1.596 (0.054)	PEPCK

Values are expressed as mean** ± **standard deviation

References for target genes [27–34]

The levels of GLUT4 and HXK-2 translational regulators rno-miR-135b-5p and rno-miR-21-5p were increased, as well as the mRNA levels of GLUT4 glucose transporter and HXK-2, which resulted in the upregulation of the levels of both proteins. However, the expression of INSR regulatory miRNAs rno-miR-194-5p and rno-miR-200a-3p altered in an opposite direction, and the mRNA level of INSR did not change significantly compared to the control, the protein expression of INSR decreased. The decreasing level of rno-miR-200a-3p and the increasing level of LEPR mRNA eventuated to significantly elevated LEPR protein expression. Both the GCK regulator rno-miR-152-3p and the IGFR1 regulator rno-miR-96-5p showed decreased levels. Meanwhile, the elevation of GCK and IGFR1 mRNA expression was observed in the hypothalamus of the STZ-treated animals compared to the controls. Finally, the elevated level of rno-miR-29a-3p showed a positive correlation with the mRNA expression level of PEPCK (Fig. 1A). Interestingly, the protein levels of the examined genes exhibited the same expression pattern as the mRNAs, even if the translational inhibitor miRNA levels were elevated or reduced. The only exception was found to be the INSR protein, which decreased at STZ treatment compared to the control hypothalamus suggesting a decreased insulin signalling cascade (Fig. 1B, C). Based on the results, it is supposed that glucose sensing, tolerance, uptake, and phosphorylation are affected not only by the miRNAs acting as regulators of glucose metabolism of the hypothalamus but maybe by additional factors e.g. inflammatory mediators or iron via modifying the expression levels of the genes related to glucose metabolism.

The miRNAs corresponding to inflammation alter the expression of fractalkine and fractalkine receptor.

Next, we examined the expression level of miRNAs related to inflammation (Table 3). The levels of rno-miR-126a-5p and rno-miR-150-5p acting on the mRNA molecules of inflammatory cytokines IL-6, IL-1β, TGFβ, and TNF-α strongly increased. Four miRNAs rno-miR-125b-5p, rno-miR-195-5p, rno-miR-29b-3p, and rno-miR-503-5p affect the translation of the inflammatory chemokine fractalkine (FKN) mRNA also showed alterations, although the first two miRNAs exhibited increasing while the latter two miRNAs showed decreasing expression levels. The rno-miR-296-3p controlling the translation of fractalkine receptor (CX3CR1) mRNA revealed a decreasing level.

**Table 3:** Changes in the relative expression of miRNAs related to inflammation in the STZ-treated rat hypothalamus

miRNA	Expression (± SD)	Target genes
rno-miR-126a-5p	1.844 (0.071)	IL-6
		IL-1β
		TGFβ
		TNF-α
rno-miR-150-5p	2.232 (0.076)	IL-6
		IL-1β
		TNF-α
rno-miR-125b-5p	1.615 (0.054)	Fractalkine
rno-miR-195-5p	1.195 (0.074)	Fractalkine
rno-miR-29b-3p	0.802 (0.041)	Fractalkine
rno-miR-503-5p	0.651 (0.038)	Fractalkine
rno-miR-296-3p	0.474 (0.032)	CX3CR1

Values are expressed as mean** ± **standard deviation

References for target genes [35–43]

**Fig. 2:**
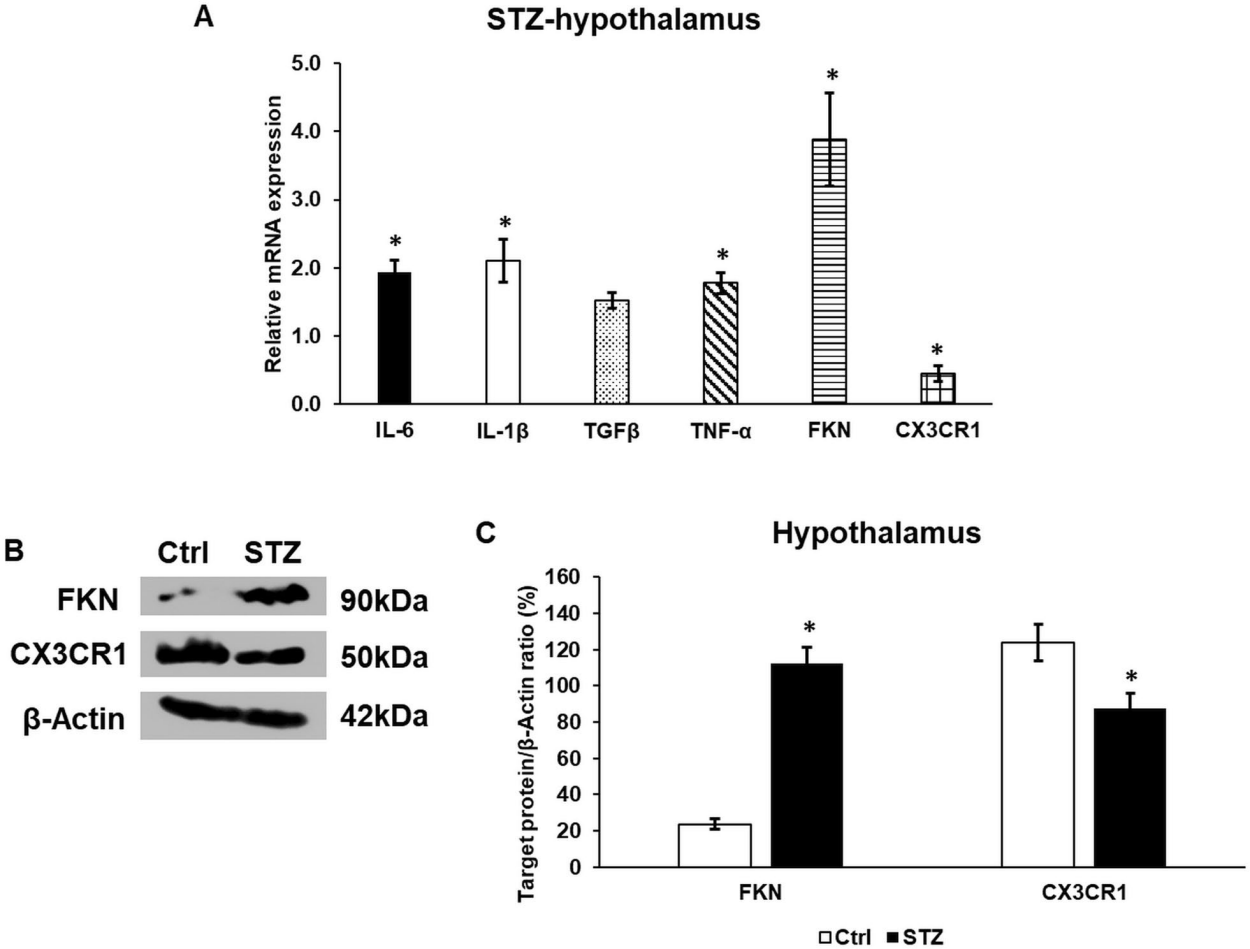
mRNA (**A**) and Western blot (**B**, **C**) analyses of the target genes of inflammationrelated miRNAs. Real time PCR was carried out using a SYBR Green protocol. β-actin was used as the housekeeping gene for the normalization of the reaction. The relative gene expression of the control hypothalamus samples was regarded as 1. Real time polymerase chain reactions were carried out in triplicate in each independent experiment. For the WB, the same amount of protein from each lysate was used for the analysis. **A** mRNA expression levels of IL-6, IL-1β, TGFβ, TNF-α, FKN, and fractalkine receptor (CX3CR1). **B** Western blot analysis of the target proteins of inflammation. **C** Optical density analysis of the target proteins. The columns represent mean values, error bars show the standard deviation (SD) of three independent determinations (n = 3). Asterisk indicates *p* < 0.05 compared to the control. IL-6: interleukin-6; IL-1β: interleukin-1β; TGFβ: transforming growth factor β; TNF-α: tumor necrosis factor-α; FKN: fractalkine; CX3CR1: fractalkine receptor; STZ: streptozotocin

According to the changes in the relative expression of miRNAs, the mRNA levels of the listed target genes were monitored (Fig. 2A). The mRNA levels of the pro-inflammatory cytokines IL-6, IL-1β, and TNF-α were significantly elevated in the hypothalamus of the STZ-treated animals suggesting the development of hypothalamic inflammation. FKN also showed significant raise, which was higher compared to the pro-inflammatory cytokines (Fig. 2A). Meanwhile, the mRNA level of CX3CR1 exhibited a reduced level (Fig. 2A). The protein expression analysis of FKN and its receptor CX3CR1 revealed the significant elevation of FKN and the decrease of CX3CR1, which followed the mRNA levels (Fig. 2B, C). These observations suggest the role of the FKN/CX3CR1 axis in the regulation of hypothalamic inflammation.

The miRNAs corresponding to iron metabolism show alterations in the STZ-treated rat hypothalamus and could modify the expression of the target genes at protein level.

Inflammation and the dysregulation of iron metabolism often appear and work together in neurological or metabolic diseases [44]. Alterations in the expression of iron metabolism related miRNAs were also observed in the hypothalamus of the STZ-treated animals (Table 4). Two miRNAs rno-miR-200a-3p and rno-miR-320-3p affecting the translation of TfR1 mRNA showed opposite changes at the expression level. The levels of ferroportin iron exporter regulator rno-miR-194-5p increased as well as the iron importer DMT-1 regulator rno-miR-19a-3p suggesting that STZ treatment modifies the iron transport of the hypothalamus. The rno-miR-214-3p controlling the protein expression of the secreted iron transport protein lactoferrin showed decreased level, while the level of rno-miR-133a-3p influencing FTL synthesis having a role in iron storage, elevated. Moreover, rno-miR-130a-3p controlling BMPR, which works as a regulator of HAMP, showed decreased level. Based on the results, it is supposed that iron transport, uptake, release, and storage are affected in the hypothalamus of the STZ-treated animals. It can be also supposed that the HAMP mRNA level is indirectly affected by the alterations found in the miRNA expression controlling the translation of HAMP transcriptional regulators.

**Table 4:** Changes in the relative expression of miRNAs related to iron metabolism in the STZ-treated rat hypothalamus

miRNA	Expression (± SD)	Target genes
rno-miR-194-5p	1.539 (0.067)	Ferroportin
rno-miR-200a-3p	0.051 (0.009)	TfR1
		Leptin receptor
rno-miR-320-3p	1.201 (0.045)	TfR1
rno-miR-19a-3p	1.309 (0.081)	DMT-1
rno-miR-214-3p	0.671 (0.061)	Lactoferrin
rno-miR-133a-3p	1.514 (0.032)	Ferritin light chain

Values are expressed as mean** ± **standard deviation

References for target genes [45–48]

**Fig. 3:**
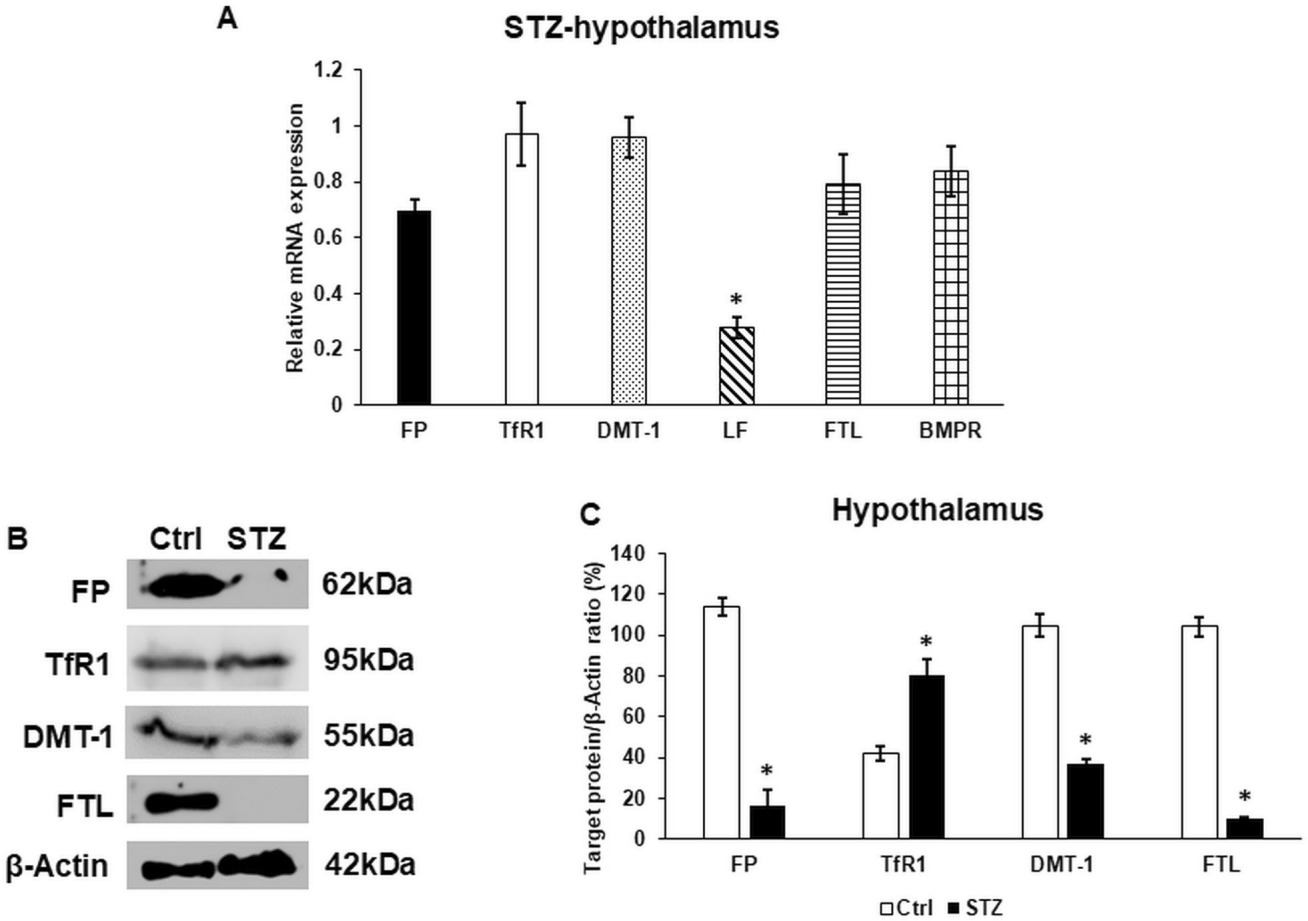
mRNA (**A**) and Western blot (**B**, **C**) analyses of the target genes of iron metabolism related miRNAs. Real time PCR was carried out using a SYBR Green protocol. β-actin was used as the housekeeping gene for the normalization of the reaction. The relative gene expression of the control hypothalamus samples was regarded as 1. Real time polymerase chain reactions were carried out in triplicate in each independent experiment. For the WB, the same amount of protein from each lysate was used for the analysis. **A** mRNA expression levels of iron metabolism related genes FP, TfR1, DMT-1, LF, FTL, and BMPR. **B** Western blot analysis of the target proteins of iron metabolism FP, TfR1, DMT-1, and FTL. **C** Optical density analysis of FP, TfR1, DMT-1 and FTL. The columns represent mean values, error bars show the standard deviation (SD) of three independent determinations (n = 3). Asterisk indicates *p* < *0.05* compared to the control. FP: ferroportin; TfR1: transferrin receptor 1; DMT-1: divalent metal transporter-1; LF: lactoferrin; FTL: ferritin light chain; BMPR: bone morphogenic protein receptor; STZ: streptozotocin

According to the changes in the relative expression of miRNAs, the mRNA levels of their target genes were determined. The mRNA expression levels of TfR1, DMT-1, FTL and BMPR did not show significant changes in the STZ-treated hypothalamus (Fig. 3A). Meanwhile, both FP and LF mRNA levels decreased after STZ treatment compared to the control hypothalamus (Fig. 3A). At protein level, FP and FTL followed the changes in the mRNA levels (Fig. 3B, C). Despite this, the DMT-1 protein level was downregulated although the mRNA level did not change due to STZ treatment (Fig. 3B, C) suggesting the inhibitory effect of miR-19a-3p on DMT-1 translation. Moreover, TfR1 protein showed elevated level, proposing that the reduction in the expression of the inhibitory miR-200a-3p triggered TfR1 translation. These observations also propose that the expression of target proteins may be influenced by additional miRNAs or other regulators such as inflammatory molecules or iron regulatory proteins (IRPs).

The mRNA and WB analyses of iron metabolism related genes show divergence in the STZ-treated hypothalamus.

**Fig. 4:**
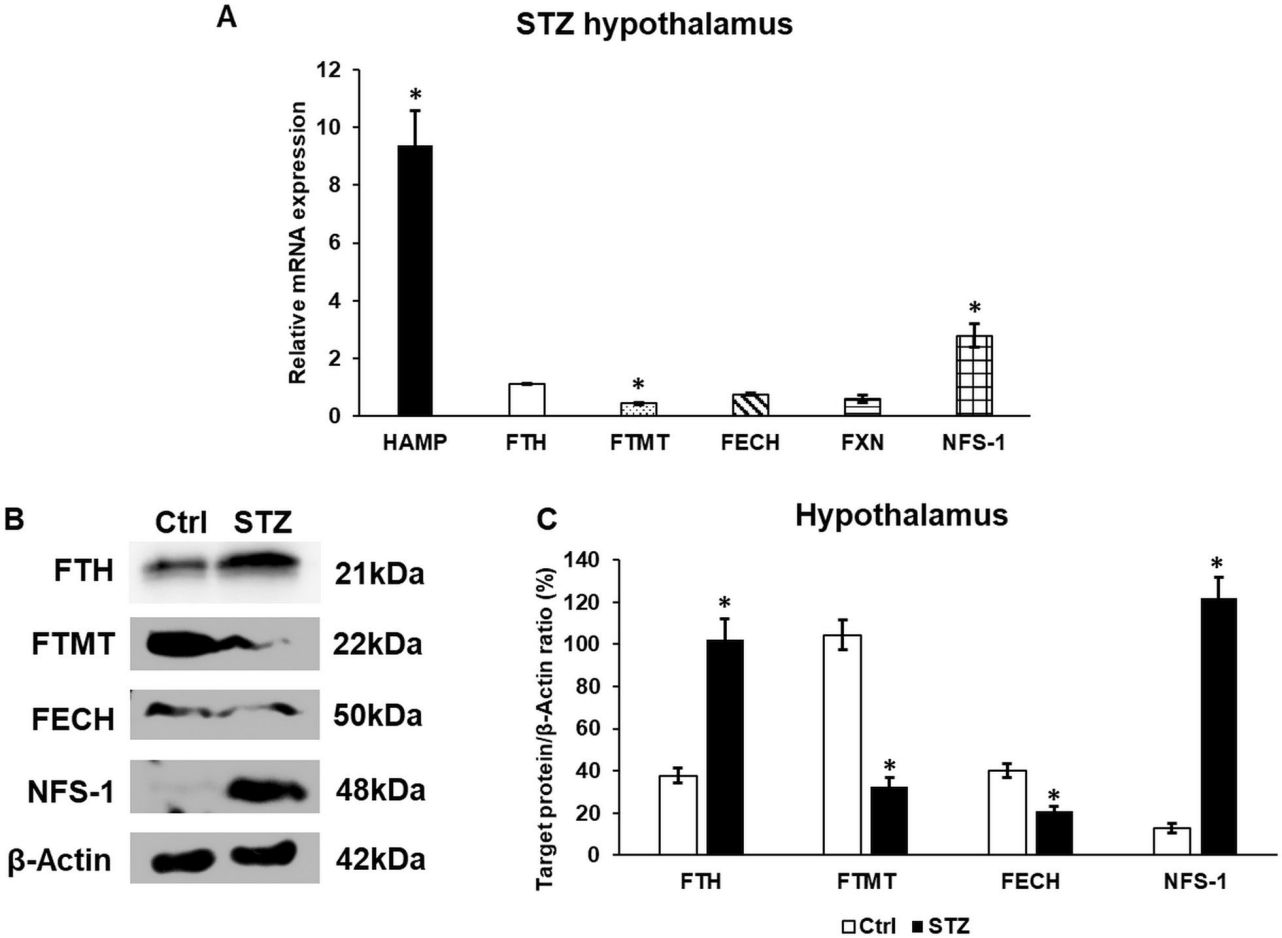
mRNA (**A**) and Western blot (**B**, **C**) analyses of the iron genes. Real time PCR was carried out using a SYBR Green protocol. β-actin housekeeping gene was used as the internal control for the normalization of the reaction. The relative gene expression of the control hypothalamus samples was considered as 1. Real time polymerase chain reactions were carried out in triplicate in each independent experiment. For the WB, the same amount of protein from each lysate was used for the analysis. **A** mRNA expression levels of iron metabolism related genes HAMP, FTH, FTMT, FECH, FXN and NFS-1. **B** Western blot analysis of the proteins of iron metabolism FTH, FTMT, FECH and NFS-1. **C** Optical density analysis of FTH, FTMT, FECH and NFS-1. The columns represent mean values, error bars show the standard deviation (SD) of three independent determinations (n = 3). Asterisk indicates *p* < *0.05* compared to the control. HAMP: hepcidin antimicrobial peptide; FTH: ferritin heavy chain; FTMT: mitochondrial ferritin; FECH: ferrochelatase; FXN: frataxin; NFS-1: cysteine desulfurase-1; STZ-streptozotocin.

The internalisation of FP and the decreasing level of FTL predispose iron retention and iron liberation from cytosolic storage, which in turn alter the iron content of the labile iron pool. Therefore, we examined the expression of HAMP, the master iron regulator, FTH, the cytosolic iron storage molecule, and FTMT acting also as iron storage protein for FECH, FXN and NFS-1, which are responsible for heme and iron-sulphur cluster syntheses in the mitochondria. The mRNA level of HAMP significantly increased in the hypothalamus of STZ-treated animals (Fig. 4A). The mRNA level of the mitochondrial iron storage protein significantly decreased, and the NFS-1 level was significantly raised compared to the untreated animals. The FTH, FXN and FECH did not change significantly (Fig. 4A). The protein level of FTH, FTMT, FECH and NFS-1 was determined by WB (Fig. 4B, C). We found that the level of FTH significantly increased, while the FTMT level was significantly downregulated in the STZ-treated hypothalamus. The protein expression of FECH decreased, albeit the NFS-1 level was significantly elevated (Fig. 4B, C). These results suggest the alteration of mitochondrial iron utilisation in diabetic conditions.

## Discussion

The CNS plays a crucial role in the regulation of glucose metabolism [2]. The hypothalamus of the CNS is implicated in the development of diabetes due to its glucose-sensing function [3]. In the hypothalamus, two types of glucose-sensing neurons can be found mainly in the hypothalamic nuclei, the arcuate (ARC) and the ventromedial (VMH) nuclei. Among the glucose-sensing neurons, the glucose-excited neurons are activated by hyperglycaemia, while hypoglycaemia activates glucose-inhibited neurons [49]. In the ARC the pro-opiomelanocortin (POMC) neurons are activated by elevated glucose level, while the neuropeptide Y/Agouti-related protein (NPY/AgRP) neurons are regulated by reduced blood glucose level [5]. VMH neurons control blood glucose level via influencing insulin secretion of the pancreas [4]. Dysregulation of the hypothalamic glucose control leads to abnormal glucose metabolism [6]. It changes the action of insulin on hepatic gluconeogenesis and causes insulin resistance in the hypothalamus that may conduct the development of peripheral insulin resistance [50]. Recent evidence has proven that the hypothalamus plays a special role in the development of diabetes mellitus [51].

We used the STZ-induced rat type 1 diabetes model [52] to investigate the action of hyperglycaemia on the glucose metabolism, inflammation, and iron metabolism related miRNAs in the hypothalamus as well as the mRNA and protein expressions of the target genes of these miRNAs. We also examined the effect of diabetic conditions on the regulation of iron homeostasis, iron transport and storage, and iron-dependent mitochondrial functions of the hypothalamus to reveal the interaction between diabetes and the disturbances of iron homeostasis.

MicroRNAs are short non-coding molecules working as key regulators of gene expression, usually by affecting the stability of the mRNA molecules. Therefore, they usually inhibit translation [20]. The role of miRNAs in the development of diabetes is under massive investigation [19]. Recent studies have described the action of several miRNA families (e.g. miR-17/92, miR143-145, miR-130, let-7, miR-200, miR-33, and miR-29) in the development of obesity and insulin resistance in adipose tissue, liver and skeletal muscle [24, 53, 54]. Hypothalamic miRNAs are supposed to have a role in the control of energy balance by modifying the expression of insulin receptor and leptin receptor acting as regulators of hypothalamic glucose metabolism [22, 23].

We found 18 miRNAs with altered expression levels in the hypothalamus of the STZ-treated animals, which act as the regulators of mRNAs involved in glucose metabolism, pro-inflammatory cytokine synthesis, and iron homeostasis suggesting a link between these processes in diabetes. The increasing level of miR-29a-3p and the elevated mRNA expression of PEPCK in the hypothalamus were correlated with the expression levels in the liver showing the systemic effect of STZ treatment (Additional file 1: Table S1, Fig. S1), which generated the development of diabetic condition [27]. The decreasing level of miR-200a-3p may influence the translation of INSR, LEPR mRNAs, as well as the iron importer TfR1 proving an interaction between diabetic condition and iron metabolism. We also found that increasing level of miR-194-5p resulted in a decreasing protein level of INSR and FP supporting another interaction with glucose and iron metabolism. The alterations in the expression level of miR-135b-5p, miR-21-5p, miR-200a-3p, miR-152-3p and miR-96-5p could modify hypothalamic glucose sensing, tolerance, uptake, and phosphorylation via affecting the stability of HXK-2, INSR, LEPR, GCK, GLUT4, IGFR1, and PEPCK mRNA molecules and as consequence miRNAs indirectly influence the protein expression levels of the aforementioned genes. The changes at protein level of HXK-2 and GCK in the hypothalamus of the STZ-treated rats predispose to enhanced glucose phosphorylation [55], while the elevated level of GLUT4 suggests that not only insulin signalling but miRNAs affected protein synthesis is also important in the expression of GLUT4 and glucose uptake. Moreover, the elevated level of inflammatory cytokines such as IL-6 can contribute to the increased GLUT4 level independently of insulin [56]. On the other hand, the raising protein level of LEPR may trigger the effect of leptin on lowering glucose concentration in the circulation [57, 58]. The level of miR-200a, known to act as an inhibitor of LEPR and INSR translation [23, 59], decreased showing a negative correlation with LEPR protein changing leptin sensitivity [60] but a positive correlation with the protein level of INSR. A possible reason for this latter result is the increased expression of an additional INSR inhibitory miRNA, miR-194-5p, leading to a decrease in the level of INSR protein. In addition, the alteration of iron metabolism especially the intracellular iron content also influences INSR expression [61]. This result proposes the decreased activity of the insulin signalling cascade [62].

Recent studies have revealed that hypothalamic inflammation contributes to the development of diabetes [9, 10, 63]. Increased activity of NFκB and JNK signalling pathways in the hypothalamus leads to the synthesis of IL-6 and TNF-α triggering neuroinflammation [64]. Hypothalamic IL-6, IL-1β and TNF-α pro-inflammatory cytokine mRNA levels were found to be positively correlated with miR-126a and miR-150 levels suggesting the activation of hypothalamic microglia and astrocytes and the development of inflammation in the STZ-treated rats. It has been described that not only cytokines, but the disruption of chemokines has an important role in the hypothalamic inflammatory process [9]. Fractalkine (FKN), which is crucial in the neuron-microglial crosstalk via the activation of microglial CX3CR1, has been implicated in the development of hypothalamic inflammation in obesity [10]. Our results show that the levels of four miRNAs, miR125b-5p, miR-195-5p, miR29b-3p and miR-503-5p were altered in the hypothalamus of the STZ-treated rats resulting in the elevation of FKN protein expression. The expression of CX3CR1 regulator miR-296-3p was found to be reduced showing a positive correlation with CX3CR1 mRNA as well as protein levels. However, reverse changes were observed in the protein expression of FKN and its receptor CX3CR1, the downstream signalling pathways may contribute to the overexpression of pro-inflammatory cytokines triggering inflammation.

Neuron-microglia communication is involved not only in the energy balance and inflammation but also in the regulation of iron homeostasis via the FKN/CX3CR1 axis [7, 17]. FKN and pro-inflammatory cytokines such as IL-6, increase cellular iron retention by elevating HAMP expression and triggering FP internalisation contributing to iron-mediated toxicity and neuronal cell death [17, 65, 66]. Our observations correlate with our previous results that the mRNA level of HAMP significantly increased, and FP was eliminated from the plasma membrane in the STZ-treated hypothalamus suggesting iron accumulation. One of the signalling pathways that may contribute to the elevated HAMP level is the BMPR/SMAD [66], also leptin/LEPR mediated signalling cascade works as a positive regulator of HAMP expression [14]. We found several miRNAs with altered expression acting as regulators of iron metabolism related genes. The miRNA, mRNA, and protein analyses of the diabetic hypothalamus revealed that the iron import via DMT-1, the iron export by FP, and the iron storage mediated by FTL were all influenced by miRNAs like miR-194-5p, miR-19a-3p, and miR-133a-3p suggesting the disturbance of hypothalamic iron homeostasis. It was revealed that the level of DMT-1 decreased suggesting the inhibitory effect of the regulatory miRNA on DMT-1 translation. Moreover, DMT-1 translation is also regulated by the intracellular iron content by IRPs; therefore, the decreasing level of DMT-1 supposes the increased intracellular iron concentration. Alternatively, Zip8 and Steap2 iron importers can take the role of DMT-1 in iron uptake of brain cells as they are not under the regulation of IRPs [67] and may contribute to the increased intracellular iron content causing iron overload. In the case of FP, the expression of regulatory miRNA showed an increasing level and the mRNA level decreased, both independent changes could contribute to the decreasing FP protein level. Indeed, we cannot exclude the effect of hepcidin on FP, but since there is no complete internalisation, the hepcidin may act in an internalisation-independent way, by inhibiting iron release via the iron exporter contributing iron retention of the cells [68, 69]. Moreover, the mRNA level of LF, a multipurpose glycoprotein, significantly decreased suggesting the deterioration of iron transport between neurons and glial cells, as well as the decreasing rate of ROS scavenging [70]. Normally, the TfR1 protein level should be downregulated by IRP by binding to the IRE element on the 3’end of the mRNA, upon intracellular iron accumulation but there are several reasons for the deregulation of iron metabolism. It has been described that TfR1 expression elevates at neuroinflammation. The possible reason for this is that inflammatory signals revoke the blocking effect of hepcidin on cellular iron uptake via TfR1, and therefore promote brain iron overload [71–73].

The deregulation of IRP1 by pro-inflammatory cytokines may also contribute to the upregulation of TFR1 protein, downregulation of FP and intracellular iron deposition [74].

Our results support the development of iron accumulation and imbalance in the hypothalamus of STZ-treated animals as the cytosolic iron oxidizing and storage protein FTH level increased but the mitochondrial form of iron storage protein FTMT decreased. The increasing level of FTH can antagonise the reactivity of the labile iron pool and prevents iron-mediated oxidative stress [75]. The reason for the differential changes in FTL and FTH protein levels is that FTH becomes more abundant at inflammation [76], which also occurs in the diabetic condition in the brain. FTH expression is also triggered by inflammation e.g. IL-1, IL-6 and TNF-α pro-inflammatory cytokines via the NFκB signalling pathway [77, 78], which is regulated by FKN showing increased levels in our experiments. Different cell types of the hypothalamus express the two ferritins in different ratio: oligodendrocytes have equal amounts of both H and L subunits, whereas microglia express L-rich ferritin, and neurons have H-subunit abundant ferritin [79]. For elucidating the exact role of the different types of hypothalamic cells in the regulation of iron metabolism in diabetes further experiments are needed.

Moreover, it seems that the mitochondrial iron utilization was also altered: the protein level of FECH responsible for iron incorporation into heme decreased while the expression of NFS-1 working in the iron-sulphur cluster synthesis raised. The iron-sulphur clusters synthesised by the mitochondria convert IRP1 to aconitase to restore the normal expression of iron transporters and maintain the tricarboxylic acid (TCA) cycle [75, 80].

## Conclusion

Based on our findings the STZ-induced diabetic condition affects hypothalamic glucose metabolism, inflammation, and iron homeostasis. The underlying molecular mechanisms mediating interactions between these processes are incompletely understood. It is supposed that they are linked together via the altered expression of common miRNAs as well as the increased expression of FKN. FKN acts as a regulator of inflammation and iron metabolism via controlling the downstream signalling cascades mediated by FKN/CX3CR1 interaction and it appears to work as a link between diabetes, inflammation, and iron metabolism. The dysregulation of hypothalamic iron homeostasis may contribute to the degeneration of neuronal cells and the imbalance of energy homeostasis. Our results raise the possibility that FKN could be a potential target of new therapies targeting both inflammation and iron disturbances in diabetic conditions.

## Supplementary Information

**Additional file 1:** Expression level of glucose metabolism-related miRNAs and mRNA analysis of the target genes in the liver of STZ-treated animals.

## Data Availability

The datasets analysed during the current study are available from the corresponding author upon reasonable request.

## Abbreviations

ARC: Arcuate nucleus
BMPR: Bone morphogenic protein receptor
CNS: Central nervous system
CX3CL1: Fractalkine
CX3CR1: Fractalkine receptor
DMT-1: Divalent metal transporter-1
FECH: Ferrochelatase
FKN: Fractalkine
FP: Ferroportin
FTH: Ferritin heavy chain
FTL: Ferritin light chain
FTMT: Mitochondrial ferritin
FXN: Frataxin
GAPDH: Glyceraldehyde-3-phospho-dehydrogenase
GCK: Glucokinase
GLUT4: Glucose transporter 4
HAMP: Hepcidin antimicrobial peptide
HXK-2: Hexokinase-2
IGFR1: Insulin-like growth factor receptor 1
IL-1β: Interleukin -1beta
IL-6: Interleukin-6
INSR: Insulin receptor
IRP1: Iron regulatory protein 1
JNK: Jun N terminal kinase
LEPR: Leptin receptor
LF: Lactoferrin
NFS-1: Cysteine desulphurase-1
NFκB: Nuclear factor kappa B
PEPCK: Phosphoenolpyruvate carboxykinase
POMC: Pro-opiomelanocortin
ROS: Reactive oxygen species
SMAD: Suppressor of Mothers against Decapentaplegic
STZ: Streptozotocin
TfR1: Transferrin receptor 1
TGFβ: Transforming growth factor beta
TNF-α: Tumour necrosis factor-alpha
VMH: Ventromedial nucleus

## References

[1] Khan MAB, Hashim MJ, King JK, Govender RD, Mustafa H, Kaabi J (2020). Epidemiology of Type 2 diabetes - Global burden of disease and forecasted trends. J Epidemiol Glob Health..

[2] Lundqvist MH, Almby K, Abrahamsson N, Eriksson JW (2019). Is the brain a key player in glucose regulation and development of type 2 diabetes?. Front Physiol.

[3] Yoon NA, Diano S (2021). Hypothalamic glucose-sensing mechanisms. Diabetologia.

[4] Varela L, Horvath TL (2012). Leptin and insulin pathways in POMC and AgRP neurons that modulate energy balance and glucose homeostasis. EMBO Rep.

[5] Fioramonti X, Contié S, Song Z, Routh VH, Lorsignol A, Pénicaud L (2007). Characterization of glucosensing neuron subpopulations in the arcuate nucleus: Integration in neuropeptide y and pro-opio melanocortin networks?. Diabetes.

[6] Koshiyama H, Hamamoto Y, Honjo S, Wada Y, Ikeda H (2006). Hypothalamic pathogenesis of type 2 diabetes. Med Hypotheses.

[7] Léon S, Nadjar A, Quarta C (2021). Microglia–neuron crosstalk in obesity: Melodious interaction or kiss of death?. Int J Mol Sci.

[8] Sheridan GK, Murphy KJ (2013). Neuron–glia crosstalk in health and disease: fractalkine and CX 3 CR1 take centre stage. Open Biol.

[9] Le Thuc O, Stobbe K, Cansell C, Nahon JL, Blondeau N, Rovère C (2017). Hypothalamic inflammation and energy balance disruptions: Spotlight on chemokines. Front Endocrinol (Lausanne)..

[10] Morari J, Anhe GF, Nascimento LF, De Moura RF, Razolli D, Solon C (2014). Fractalkine (CX3CL1) is involved in the early activation of hypothalamic inflammation in experimental obesity. Diabetes.

[11] Simcox JA, McClain DA (2013). Iron and diabetes risk. Cell Metab.

[12] Rajpathak SN, Crandall JP, Wylie-Rosett J, Kabat GC, Rohan TE, Hu FB (2009). The role of iron in type 2 diabetes in humans. Biochim Biophys Acta Gen Subj.

[13] Liu J, Li Q, Yang Y, Ma L (2020). Iron metabolism and type 2 diabetes mellitus: A meta-analysis and systematic review. J Diabetes Investig.

[14] Kimita W, Bharmal SH, Ko J, Cho J, Petrov MS (2021). Relationship between energy balance and circulating levels of hepcidin and ferritin in the fasted and postprandial states. Nutrients.

[15] Derghal A, Astier J, Sicard F, Couturier C, Landrier JF, Mounien L (2019). Leptin modulates the expression of mirnas-targeting POMC mRNA by the JAK2-STAT3 and PI3K-akt pathways. J Clin Med.

[16] Fernández-Real JM, López-Bermejo A, Ricart W (2002). Cross-talk between iron metabolism and diabetes. Diabetes.

[17] Pandur E, Tamási K, Pap R, Varga E, Miseta A, Sipos K (2019). Fractalkine induces hepcidin expression of BV-2 Microglia and Causes Iron Accumulation in SH-SY5Y Cells. Cell Mol Neurobiol.

[18] Martins AC, Almeida JI, Lima IS, Kapitão AS, Gozzelino R (2017). Iron Metabolism and the Inflammatory Response. IUBMB Life.

[19] Jean-François L, Derghal A, Mounien L (2019). Micrornas in obesity and related metabolic disorders. Cells.

[20] Bartel DP (2004). MicroRNAs: genomics, biogenesis, mechanism, and function. Cell.

[21] Davis M, Clarke S (2013). Influence of microRNA on the maintenance of human iron metabolism. Nutrients.

[22] Li RJW, Zhang SY, Lam TKT (2020). Interaction of glucose sensing and leptin action in the brain. Mol Metab..

[23] Schneeberger M, Gomez-Valadés AG, Ramirez S, Gomis R, Claret M (2015). Hypothalamic miRNAs: Emerging roles in energy balance control. Front Neurosci.

[24] Deiuliis JA (2016). MicroRNAs as regulators of metabolic disease: Pathophysiologic significance and emerging role as biomarkers and therapeutics. Int J Obes.

[25] Paxinos G, Watson C. The Rat Brain in Stereotaxic Coordinates: Hard Cover Edition. 7th Editio. Elsevier Science; 2013.

[26] ImageJ. https://imagej.nih.gov/ij/index.html

[27] Pandey AK, Verma G, Vig S, Srivastava S, Srivastava AK, Datta M (2011). MiR-29a levels are elevated in the db/db mice liver and its overexpression leads to attenuation of insulin action on PEPCK gene expression in HepG2 cells. Mol Cell Endocrinol.

[28] Xu C, Niu JJ, Zhou JF, Wei YS (2019). MicroRNA-96 is responsible for sevoflurane-induced cognitive dysfunction in neonatal rats via inhibiting IGF1R. Brain Res Bull.

[29] Ofori JK, Salunkhe VA, Bagge A, Vishnu N, Nagao M, Mulder H (2017). Elevated miR-130a/miR130b/miR-152 expression reduces intracellular ATP levels in the pancreatic beta cell. Sci Rep.

[30] Crépin D, Benomar Y, Riffault L, Amine H, Gertler A, Taouis M (2014). The over-expression of miR-200a in the hypothalamus of ob/ob mice is linked to leptin and insulin signaling impairment. Mol Cell Endocrinol.

[31] Ling HY, Hu B, Hu XB, Zhong J, Feng SD, Qin L (2012). MiRNA-21 reverses high glucose and high insulin induced insulin resistance in 3T3-L1 adipocytes through targeting phosphatase and tensin homologue. Exp Clin Endocrinol Diabetes.

[32] Latouche C, Natoli A, Reddy-Luthmoodoo M, Heywood SE, Armitage JA, Kingwell BA (2016). MicroRNA-194 modulates glucose metabolism and its skeletal muscle expression is reduced in diabetes. PLoS ONE.

[33] Yang Y, IshakGabra MB, Hanse EA, Lowman XH, Tran TQ, Li H (2019). MiR-135 suppresses glycolysis and promotes pancreatic cancer cell adaptation to metabolic stress by targeting phosphofructokinase-1. Nat Commun.

[34] Wang H (2020). Microrna, diabetes mellitus and colorectal cancer. Biomedicines.

[35] Luo W, Lin Y, Meng S, Guo Y, Zhang J, Zhang W (2016). MiRNA-296–3p modulates chemosensitivity of lung cancer cells by targeting CX3CR1. Am J Transl Res..

[36] Zhou R, Gong AY, Chen D, Miller RE, Eischeid AN, Chen XM (2013). Histone Deacetylases and NF-kB Signaling Coordinate Expression of CX3CL1 in Epithelial Cells in Response to Microbial Challenge by Suppressing miR-424 and miR-503. PLoS ONE.

[37] Fenn AM, Smith KM, Lovett-Racke AE, Guerau-de-Arellano M, Whitacre CC, Godbout JP (2013). Increased micro-RNA 29b in the aged brain correlates with the reduction of insulin-like growth factor-1 and fractalkine ligand. Neurobiol Aging.

[38] Mao M, Xu Y, Zhang XY, Yang L, An XB, Qu Y (2020). MicroRNA-195 prevents hippocampal microglial/macrophage polarization towards the M1 phenotype induced by chronic brain hypoperfusion through regulating CX3CL1/CX3CR1 signaling. J Neuroinflam.

[39] Batool A, Wang YQ, Hao XX, Chen SR, Liu YX (2018). A miR-125b/CSF1-CX3CL1/tumor-associated macrophage recruitment axis controls testicular germ cell tumor growth. Cell Death Dis.

[40] Chen S, Zhu H, Sun J, Zhu L, Qin L, Wan J (2021). Anti-inflammatory effects of miR-150 are associated with the downregulation of STAT1 in macrophages following lipopolysaccharide treatment. Exp Ther Med.

[41] Chen J, Cui C, Yang X, Xu J, Venkat P, Zacharek A (2017). MiR-126 affects brain-heart interaction after cerebral ischemic stroke. Transl Stroke Res.

[42] Huang J, Zhu L, Qiu C, Xu X, Zhang L, Ding X (2017). MicroRNA miR-126–5p enhances the inflammatory responses of monocytes to lipopolysaccharide stimulation by suppressing cylindromatosis in chronic HIV-1 Infection. J Virol.

[43] Chen Y-J, Lin T-L, Cai Z, Yan C-H, Gou S-R, Zhuang Y-D (2020). Assessment of acute pancreatitis severity via determination of serum levels of hsa-miR-126-5p and IL-6. Exp Ther Med.

[44] Vela D (2018). The dual role of hepcidin in brain iron load and inflammation. Front Neurosci.

[45] Liao Y, Du X, Lönnerdal B (2010). miR-214 regulates lactoferrin expression and pro-apoptotic function in mammary epithelial cells. J Nutr.

[46] Yujing Li ZS (2013). The Crosstalk between Micro RNA and Iron Homeostasis. Int J Genomic Med..

[47] Schaar DG, Medina DJ, Moore DF, Strair RK, Ting Y (2009). miR-320 targets transferrin receptor 1 (CD71) and inhibits cell proliferation. Exp Hematol.

[48] Hamara K, Bielecka-Kowalska A, Przybylowska-Sygut K, Sygut A, Dziki A, Szemraj J (2013). Alterations in expression profile of iron-related genes in colorectal cancer. Mol Biol Rep.

[49] Routh VH (2010). Glucose sensing neurons in the ventromedial hypothalamus. Sensors (Switzerland).

[50] Pocai A, Obici S, Schwartz GJ, Rossetti L (2005). A brain-liver circuit regulates glucose homeostasis. Cell Metab.

[51] Jais A, Brüning JC (2022). Arcuate nucleus-dependent regulation of metabolism-pathways to obesity and diabetes mellitus. Endocr Rev.

[52] Furman BL (2015). Streptozotocin-Induced Diabetic Models in Mice and Rats. Curr Protoc Pharmacol.

[53] Fu X, Dong B, Tian Y, Lefebvre P, Meng Z, Wang X (2015). MicroRNA-26a regulates insulin sensitivity and metabolism of glucose and lipids. J Clin Invest.

[54] Kristensen MM, Davidsen PK, Vigelsø A, Hansen CN, Jensen LJ, Jessen N (2017). miRNAs in human subcutaneous adipose tissue: Effects of weight loss induced by hypocaloric diet and exercise. Obesity.

[55] Rabbani N, Thornalley PJ (2019). Hexokinase-2 glycolytic overload in diabetes and ischemia-reperfusion injury. Trends Endocrinol Metab.

[56] Marko DM, Foran G, Vlavcheski F, Baron DC, Hayward GC, Baranowski BJ (2020). Interleukin-6 Treatment Results in GLUT4 Translocation and AMPK Phosphorylation in Neuronal SH-SY5Y Cells. Cells.

[57] Meek TH, Morton GJ (2016). The role of leptin in diabetes: metabolic effects. Diabetologia.

[58] Pandit R, Beerens S, Adan RAH (2017). Role of leptin in energy expenditure: The hypothalamic perspective. Am J Physiol - Regul Integr Comp Physiol.

[59] Pandey P, Seshadri V (2014). Role of miR-200a in regulating the insulin signalling in the hypothalamus. Non-coding RNAs Endocrinol..

[60] Derghal A, Djelloul M, Azzarelli M, Degonon S, Tourniaire F, Landrier JF (2018). MicroRNAs are involved in the hypothalamic leptin sensitivity. Epigenetics.

[61] Fargion S, Dongiovanni P, Guzzo A, Colombo S, Valenti L, Fracanzani AL (2005). Iron and insulin resistance. Aliment Pharmacol Ther Suppl..

[62] Chen Y, Huang L, Qi X, Chen C (2019). Insulin receptor trafficking: Consequences for insulin sensitivity and diabetes. Int J Mol Sci.

[63] Rahman MH, Bhusal A, Lee WH, Lee IK, Suk K (2018). Hypothalamic inflammation and malfunctioning glia in the pathophysiology of obesity and diabetes: Translational significance. Biochem Pharmacol.

[64] Cai D (2012). One step from prediabetes to diabetes: Hypothalamic inflammation?. Endocrinology.

[65] D’Mello SR, Kindy MC (2020). Overdosing on iron: Elevated iron and degenerative brain disorders. Exp Biol Med.

[66] Varga E, Pap R, Jánosa G, Sipos K, Pandur E (2021). IL-6 regulates hepcidin expression via the BMP/SMAD pathway by altering BMP6, TMPRSS6 and TfR2 expressions at normal and inflammatory conditions in BV2 microglia. Neurochem Res.

[67] Ji C, Kosman DJ (2015). Molecular mechanisms of non-transferrin-bound and transferring-bound iron uptake in primary hippocampal neurons. J Neurochem.

[68] Aschemeyer S, Qiao B, Stefanova D, Valore EV, Sek AC, Alex Ruwe T (2018). Structure-function analysis of ferroportin defines the binding site and an alternative mechanism of action of hepcidin. Blood.

[69] Zhang DL, Rouault TA (2018). How does hepcidin hinder ferroportin activity?. Blood.

[70] Li YQ, Guo C (2021). A review on lactoferrin and central nervous system diseases. Cells.

[71] Vela D (2018). The dual role of hepcidin in brain iron load and inflammation. Front Neurosci.

[72] Urrutia P, Aguirre P, Esparza A, Tapia V, Mena NP, Arredondo M (2013). Inflammation alters the expression of DMT1, FPN1 and hepcidin, and it causes iron accumulation in central nervous system cells. J Neurochem.

[73] Du F, Qian ZM, Luo Q, Yung WH, Ke Y (2015). Hepcidin suppresses brain iron accumulation by downregulating iron transport proteins in iron-overloaded rats. Mol Neurobiol.

[74] Wang J, Song N, Jiang H, Wang J, Xie J (2013). Pro-inflammatory cytokines modulate iron regulatory protein 1 expression and iron transportation through reactive oxygen/nitrogen species production in ventral mesencephalic neurons. Biochim Biophys Acta Mol Basis Dis.

[75] Zhang KH, Tian HY, Gao X, Lei WW, Hu Y, Wang DM (2009). Ferritin heavy chain-mediated iron homeostasis and subsequent increased reactive oxygen species production are essential for epithelial-mesenchymal transition. Cancer Res.

[76] Kernan KF, Carcillo JA (2017). Hyperferritinemia and inflammation. Int Immunol.

[77] Torti FM, Torti SV (2002). Regulation of ferritin genes and protein. Blood.

[78] Pandur E, Varga E, Tamási K, Pap R, Nagy J, Sipos K (2019). Effect of inflammatory mediators lipopolysaccharide and lipoteichoic acid on iron metabolism of differentiated SH-SY5Y cells alters in the presence of BV-2 microglia. Int J Mol Sci.

[79] Moos T, Morgan EH (2004). The metabolism of neuronal iron and its pathogenic role in neurological disease review. Ann N Y Acad Sci.

[80] Read AD, Bentley RE, Archer SL, Dunham-Snary KJ (2021). Mitochondrial iron–sulfur clusters: Structure, function, and an emerging role in vascular biology: Mitochondrial Fe-S Clusters – a review. Redox Biol.

