# Supplementary material for: Alterations of the expression levels of glucose, inflammation, and iron metabolism related miRNAs and their target genes in the hypothalamus of STZ-induced rat diabetes model
Source: Diabetol Metab Syndr. 2022 Oct 10;14:147. doi: 10.1186/s13098-022-00919-5 (PMC9549668; doi:10.1186/s13098-022-00919-5)
Supplement: Supplementary file 1 — Additional file 1: Expression level of glucose metabolism-related miRNAs and mRNA analysis of the target genes in the liver of STZ-treated animals. [file 13098_2022_919_MOESM1_ESM.pdf]

Alterations of the expression levels of glucose and iron metabolism related miRNAs and their target genes in the hypothalamus of STZ-induced rat diabetes model

Edina Pandur[1*], István Szabó[2], Edina Hormay[2], Ramóna Pap[1], Attila Almási[3], Katalin Sipos[1], Viktória Farkas[1], Zoltán Karádi[2]

[1]Department of Pharmaceutical Biology, Faculty of Pharmacy, University of Pécs, H-7624, Pécs, Rókus u. 4., Hungary

[2]Institute of Physiology, Medical School, University of Pécs, H-7624, Pécs, Szigeti út 12., Hungary

[3]Institute of Pharmaceutical Chemistry, Faculty of Pharmacy, University of Pécs, H-7624, Pécs, Rókus u. 4., Hungary

*Correspondence: edina.pandur@aok.pte.hu

Supplementary Table 1. Expression level of glucose metabolism-related miRNAs

miRNA	Expression (±SD)	Target genes
rno-miR-135b-5p	1.205 (0.044)	GLUT4<br>Hexokinase 2
rno-miR-194-5p	0.606 (0.022)	Insulin receptor
rno-miR-21-5p	1.085 (0.031)	GLUT4
rno-miR-200a-3p	0.107 (0.011)	Leptin receptor<br>Insulin receptor
rno-miR-152-3p	0.689 (0.021)	Glucokinase
rno-miR-96-5p	0.072 (0.023)	IGF1R
rno-miR-29a-3p	1.476 (0.049)	PEPCK

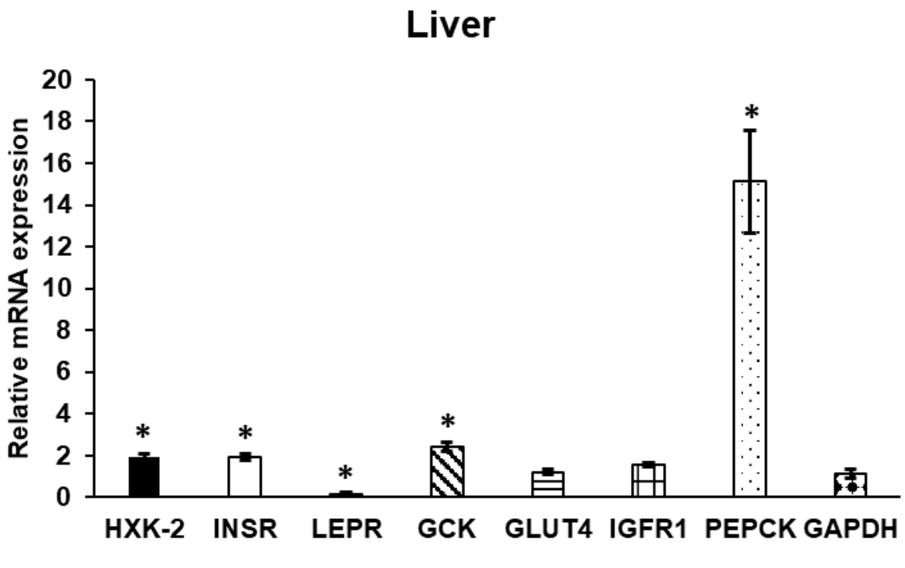

Supplementary figure 1. mRNA analysis of the target genes of glucose metabolism related miRNAs. Real time PCR was carried out using a SYBR Green protocol. β-actin housekeeping gene was used as

internal control for the normalization of the reaction. The relative gene expression of the control liver samples was regarded as 1. Real time polymerase chain reactions were carried out in triplicate in each independent experiment. The columns represent mean values, error bars show standard deviation (SD) of three independent determinations (n = 3). Asterisk indicates $p < 0.05$ compared to the control. Abbreviations: HXK-2-hexokinase-2; INSR-insulin receptor; LEPR-leptin receptor; GCK-glucokinase; GLUT4-glucose transporter 4; IGFR1-insulin-like growth factor receptor 1; PEPCK-phosphoenolpyruvate carboxykinase; GAPDH- glyceraldehyde 3-phosphate dehydrogenase; STZ-streptozotocin.
